# Auditory T-Complex Reveals Reduced Neural Activities in the Right Auditory Cortex in Musicians With Absolute Pitch

**DOI:** 10.3389/fnins.2019.00809

**Published:** 2019-08-06

**Authors:** Masato Matsuda, Hironaka Igarashi, Kosuke Itoh

**Affiliations:** Center for Integrated Human Brain Science, Brain Research Institute, Niigata University, Niigata, Japan

**Keywords:** music training, language, auditory evoked cortical potentials, brain maturation and development, balanced non-cephalic electrode

## Abstract

Absolute pitch (AP) is the ability to identify the pitch names of arbitrary musical tones without being given a reference pitch. The acquisition of AP typically requires early musical training, the critical time window for which is similar to that for the acquisition of a first language. This study investigated the left–right asymmetry of the auditory cortical functions responsible for AP by focusing on the T-complex of auditory evoked potentials (AEPs), which shows morphological changes during the critical period for language acquisition. AEPs evoked by a pure-tone stimulus were recorded in high-AP musicians, low-AP musicians, and non-musicians (*n* = 19 each). A balanced non-cephalic electrode (BNE) reference was used to examine the left–right asymmetry of the N1a and N1c components of the T-complex. As a result, a left-dominant N1c was observed only in the high-AP musician group, indicating “AP negativity,” which has previously been described as an electrophysiological marker of AP. Notably, this hemispheric asymmetry was due to a diminution of the right N1c rather than enhancement of the left N1c. A left-dominant N1a was found in both musician groups, irrespective of AP. N1c and N1a exhibited no left–right asymmetry in non-musicians. Hence, music training and the acquisition of AP are both accompanied by a left-dominant hemispheric specialization of auditory cortical functions, as indexed by N1a and N1c, respectively, but the N1c asymmetry in AP possessors was due to reduced neural activities in the right hemisphere. The use of a BNE is recommended for evaluating these radially oriented components of the T-complex.

## Introduction

Absolute pitch (AP) is the ability to identify pitch names of arbitrary musical tones without being given a reference pitch (Miyazaki, 1988, 1990; Takeuchi and Hulse, 1993). In addition to a possible genetic predisposition (Baharloo et al., 2000), the acquisition of AP usually requires early musical training (<7 years of age; Miyazaki, 1988; Takeuchi and Hulse, 1993; Miyazaki and Ogawa, 2006), the critical period similar to acquisition of a first language (Newport, 1990; Kuhl, 2000; Russo et al., 2003; Vitouch, 2003; Deutsch et al., 2006). Specialized neural circuitries that underpin AP are hypothesized to be established through early musical training (Pantev et al., 1998; Ohnishi et al., 2001), the timing of which is coincident with the maturation of language-related brain functions (Krashen, 1973; Newport, 1990; Kuhl, 2000). These parallels between AP and language are consistent with the view that AP is essentially a verbal function for labeling pitches with their names (Itoh et al., 2005).

Cerebral hemispheric asymmetry is a hallmark of language, and also of AP. AP possessors show left-dominant neural responses to both speech and music stimuli (Ohnishi et al., 2001; Itoh et al., 2005; Schulze et al., 2009, 2013; Wilson et al., 2009; Oechslin et al., 2010b), whereas non-possessors typically show left-dominant neural activities for speech stimuli alone. The geometric location of electromagnetic sources of neural responses to musical pitches are asymmetrically localized in AP possessors but symmetrically localized in non-possessors (Hirata et al., 1999). Functional connectivity in the left superior temporal gyrus is enhanced in AP possessors (Loui et al., 2012). The fractional anisotropy of the superior longitudinal fasciculus, which is a major cortical fiber that is crucial for language and music functions, is left-dominant in musicians with AP, whereas it is symmetrical in musicians with relative pitch (Oechslin et al., 2010a). Volumetric studies have also shown that AP is associated with greater left-dominant asymmetry of gray matter volume of the perisylvian brain areas (Schlaug et al., 1995; Keenan et al., 2001; Ohnishi et al., 2001; Wilson et al., 2009), which was due to either a reduction of the gray matter volume in the right hemisphere (Schlaug et al., 1995; Keenan et al., 2001; Wilson et al., 2009), or its increase in the left hemisphere (Zatorre et al., 1998). However, other studies have reported right-dominant neural activities (Hirose et al., 2005; Wengenroth et al., 2014; Burkhard et al., 2019), increased right auditory cortical volume (Wengenroth et al., 2014), or higher myelination associated with greater functional connectivity in the right auditory cortex (Kim and Knösche, 2016, 2017) in AP possessors. Integrating all these findings into a coherent hypothesis remains difficult. Nevertheless, the available evidence is consistent with the view that the typical hemispheric specialization of cerebral cortical functions for auditory processing is altered in AP possessors, the details of which remain to be elucidated.

This study analyzed the left–right asymmetry of the auditory cortical functions in AP possessors by focusing on the T-complex of auditory evoked potentials (AEPs). The T-complex is a subcomponent of the auditory N1 response, which has multiple generators in the auditory and other brain regions (Näätänen and Picton, 1987). Regarding the auditory cortical generators, two categories of sources can be distinguished on their dipole orientation, tangential or radial (Scherg et al., 1989; Woods, 1995). The tangential sources are located on the superior temporal plane, and the generated electrical potentials project vertically to the fronto-central scalp: the N1b (∼100 ms), or the vertex N1, is the most representative example. The radial sources are located on the lateral surface of the temporal lobe, and therefore, the electrical potentials project laterally to the temporal scalp. The T-complex, which is the focus of this study, is a radial component that consists of three peaks that are recorded over the temporal scalp: N1a (75–95 ms), Ta (100–115 ms), and N1c (130–170 ms) (Näätänen and Picton, 1987; Woods, 1995). The sources of T-complex have been estimated in the secondary or higher auditory cortex of the superior temporal gyrus (Scherg et al., 1989; Woods, 1995; Shahin et al., 2003).

The T-complex is a potentially promising index for investigating the hemispheric asymmetry of auditory cortical functions in AP possessors. First, as these potentials originate from radially oriented sources in the temporal lobe, the waves recorded over the left and right temporal scalp reflect neural activities in the left and right auditory cortices, respectively, (Knight et al., 1988). Clear separation of left and right neural activities would be difficult with the tangential components of AEP that are distributed maximally along the midline, such as the N1b. Second, and more importantly, the waveform of the T-complex shows morphological changes during the critical period of language acquisition (Pang and Taylor, 2000; Ponton et al., 2002; Tonnquist-Uhlen et al., 2003; Rinker et al., 2017), which likely reflect maturational changes in auditory cortical functions, and it is also affected by music training in childhood (Shahin et al., 2003). Hence, the functional hemispheric asymmetry for AP may manifest as an altered morphology of the T-complex.

In fact, a previously identified electrophysiological marker of AP, or “AP negativity” (Itoh et al., 2005), closely resembles the N1c component of the T-complex in terms of polarity, amplitude, latency, and scalp distribution. AP negativity is a negative AEP of approximately 1 μV in amplitude that peaks at approximately 150 ms in latency over the posterior temporal scalp (when recorded with a linked earlobe reference). Its amplitude is greater (i.e., more negative) in the left hemisphere, in accordance with the reported left-dominant pitch processing in AP (Brancucci et al., 2009; Wilson et al., 2009). However, the original study by Itoh et al. (2005) had several issues. First, a linked earlobe reference was used, which was not optimal for evaluating the T-complex; electric potentials from radially oriented sources could have “activated” the earlobe electrodes and thus might have altered the wave morphology. Second, the number of trials for averaging was relatively small (90 trials) for evaluating the small potential of approximately 1 μV. Furthermore, no subsequent study has yet replicated the elicitation of AP negativity in possessors of AP, to the best of our knowledge.

Therefore, we examined the morphology of the T-complex in AP possessors with a particular focus on the left–right asymmetry of the N1c component with an appropriate study design. Many trials (*n* = 300) were used to record the T-complex and evaluate its amplitudes with high reliability. To obviate the confounding effects of general music training, three groups of participants were used: musicians with high levels of AP (high-AP musicians), musicians with low-levels of AP (low-AP musicians), and non-musicians. Comparisons between the high-AP and low-AP musicians would delineate the effects of AP, whereas comparisons of the two musician groups with the non-musicians would identify the general effects of music training that are not specific to AP.

Furthermore, and quite important, a balanced non-cephalic electrode (BNE; Stephenson and Gibbs, 1951) was employed as the reference for evaluating the AEP amplitudes. Since no electrodes placed on the head (including those on the earlobes) can be assumed to be ‘quiet’ with respect to cortical potentials, measurements of AEP amplitude are always confounded by neural activities recorded at the cephalic reference channels. This poses a serious problem when the mastoid or earlobe electrodes are used as a reference (or a part of the reference) to evaluate the T-complex, because the radial sources of the T-complex are expected to “activate” these reference channels. The BNE method ameliorates this problem by using a non-cephalic reference that is placed outside the head. Contaminations of the non-cephalic channel by electrocardiograms (ECGs) are eliminated by pairing two electrodes that are placed anteriorly and posteriorly at the base of the neck. As the ECGs recorded from these electrodes have an approximately the same magnitude but with opposite polarities (with respect to the scalp), the cardiac signals can be canceled by balancing the electrical resistance between these two non-cephalic electrodes. This is the first experiment to use the BNE reference to record electroencephalograms (EEGs) in AP possessors, to our knowledge.

## Materials and Methods

### Participants

Fifty-seven healthy undergraduate students (age: 18–26 years, 15 males) participated in this study, after providing written informed consent. The participants were predominantly females, because subjects with AP were more readily available to us in this gender. This was a limitation of the study, as there is some evidence for gender differences in hemispheric lateralization related to music and AP (Luders et al., 2004; Merrett et al., 2013). Nevertheless, our results were apparently not affected by the gender of the participants (see “Results”). All participants were right-handed, as confirmed using the Edinburgh Handedness Inventory (Oldfield, 1971).

The participants were categorized into three groups according to their levels of music training and AP ability: high-AP musicians (*n* = 19, 3 males), low-AP musicians (*n* = 19, 5 males), and non-musicians (*n* = 19, 7 males). All participants had received basic-level music education as one of the curriculum requirements in Japan. Therefore, the level of music training in this study was defined in terms of the number of years in music training given by professional music teachers outside standard school education. With this definition, the average (± standard deviation) years in music training was 15.2 (± 2.0) in the high-AP group and 13.8 (± 3.2) in the low-AP group; the difference was not significant (*t* = 1.5, degrees of freedom (*df*) = 36, *P* > 0.05). The age of commencement of music training was also comparable between the high-AP musicians (4.3 ± 1.2 years old) and the low-AP musicians (4.9 ± 1.5 years old; *t* = 1.4, *df* = 36, *P* > 0.05). The non-musicians had received less than 5 years of music training, and the average was 0.9 (± 1.5) years.

The participant’s AP ability was evaluated using a previously described pitch naming test (Itoh et al., 2005), in which they were instructed to identify pitch classes (e.g., C, C#, and D, without distinguishing octaves) of 60 random piano tones covering five octaves. The AP test was essentially identical to the AP test developed by Miyazaki (1988), which has been validated by many experiments (e.g., Miyazaki, 1990; Itoh et al., 2003, 2005; Miyazaki and Ogawa, 2006; Miyazaki et al., 2012; Itoh and Nakada, 2018). Critical steps were taken to prevent the use of relative pitch. First, no reference tone was presented at any point of the test, and feedback on the accuracy of the participant’s responses was not provided. Therefore, the participants had to use their own internal long-term memory to correctly identify the pitch class. Second, the sequence of the test tones was randomized to make the use of relative pitch difficult. Finally, the inter-trial interval was set relatively short. The test sounds were presented every 5 s and the duration of sounds was approximately 1 s long; therefore, the notes had to be identified within a response time window of approximately 4 s. The criterion for high-AP musicians was 90% correct or higher, and for low-AP musicians, it was 40% or lower.

This study conformed to The Code of Ethics of the World Medical Association (Declaration of Helsinki), and was conducted in accordance with the human research guidelines of the Internal Review Board of the University of Niigata.

### Stimuli and Procedure

The stimulus was a sinusoidal tone of 1046.5 Hz, which corresponded to the frequency of C6 (American notation). A single, fixed frequency was used, because the use of multiple frequencies introduces pitch changes, neural responses to which might confound the results by the mechanisms of neural adaptation and/or mismatch negativity (Tervaniemi et al., 1993; Elmer et al., 2015; Rogenmoser et al., 2015; Greber et al., 2018). With a repeated presentation of the note, the participants could perceive it as the keynote of the stimulus sequence, but it required AP to identify the specific note as we did not provide any information about the stimulus. The sound had a duration of 350 ms (10-ms rise-time and 50-ms fall-time). The stimulus was presented monaurally (left-ear and right-ear) or binaurally in a random order via air-conduction insert earphones (Eartone 3A, Etymotic Research, Elk Grove Village, IL, United States) with a stimulus intensity of 65 dB SL. A total of 900 tones (300 tones for each ear condition) were presented using randomly varying stimulus onset asynchrony in the range of 1700–1900 ms (mean: 1800 ms). Only binaural AEPs were analyzed in this study, as it is more natural than monaural listening situations; we seldom receive completely lateralized monaural stimulations while listening to music. The monaural data will be analyzed in a subsequent paper.

The participants sat in a comfortable chair in a temperature-controlled and sound-attenuated room during the EEG recordings. They listened to the sounds passively while playing a video game (Nintendo DS, Nintendo Co., Ltd., Kyoto, Japan) to maintain wakefulness and prevent the participants from explicitly labeling the tones.

### EEG Recording and Analysis

Twenty-two Ag electrodes were applied to the scalp of each participant according to the international 10–20 system (Jasper, 1958), and the electrodes were positioned at Fp1, Fp2, Fz, F3, F4, F7, F8, Cz, C3, C4, T3, T4, CPz, Pz, P3, P4, T5, T6, O1, O2, and the left and right earlobes. Horizontal and vertical electro-oculograms (EOGs) were also recorded. In addition, two electrodes were placed on the right sternoclavicular junction and the tip of the seventh cervical spine for recording BNE data (Stephenson and Gibbs, 1951). All electrodes were referenced to CPz while collecting data. The EEGs and EOGs were amplified by a SynAmp amplifier (Neuroscan Labs, El Paso, TX, United States) with 16-bit resolution, a gain of 5000, and an analog–digital conversion rate of 10 kHz, band-passed between 0.05 and 2000 Hz. The electrode’s impedance was kept below 5 kΩ.

After data acquisition, the EEG data were downsampled to 1 kHz, re-referenced to the BNE, segmented (from −100 to 200 ms relative to the stimulus onset), and baseline-corrected to the pre-stimulus period average. The segmented data were checked for artifacts using a threshold criterion of ± 100 μV using the Fp1, Fp2, F7, and F8, and horizontal and vertical EOG channels to remove ocular artifacts. The non-rejected data were averaged and time-locked to the stimulus onset to obtain AEPs for each participant, which were low-pass filtered at 50 Hz (48 dB/oct). The number of non-rejected data segments was in the range of 224–288 (mean 262) in the high-AP musicians, 223–296 (mean 267) in the low-AP musicians, and 220–300 (mean 267) in the non-musicians. Finally, the AEPs were averaged across participants to obtain grand average waveforms.

The peak amplitudes of N1a and N1c at the bilateral temporal electrode sites (T3 and T4) were analyzed. The N1b measured at the central electrode site (Cz) was also analyzed for comparison. The Ta component was not analyzed because it was small in amplitude and difficult to measure. The grand average waveforms were used to define the time windows for N1a (70–80 ms at T3, 73–83 ms at T4), N1b (77–97 ms) and N1c (112–132 ms at T3, 119–139 ms at T4), and the peaks were defined as the most negative point in these time slots. These time windows were centered at the peak latency of the components as identified in the grand average waveforms and had a time width of 10 ms for N1a, and 20 ms for N1b and N1c.

The peak amplitudes of N1a and N1c were analyzed using a mixed-design two-way analysis of variance (ANOVA) with the group (high-AP musicians, low-AP musicians, and non-musicians) as the between-subjects factor and the hemisphere (T3 and T4) as the within-subjects factor. For the N1b amplitude, a one-way ANOVA with the group as the between-subjects factor was performed. An alpha level of *P* = 0.05 was used as the significance criterion. *P*-values were corrected for multiple comparisons by using the Bonferroni method wherever appropriate.

## Results

**FIGURE 1:**
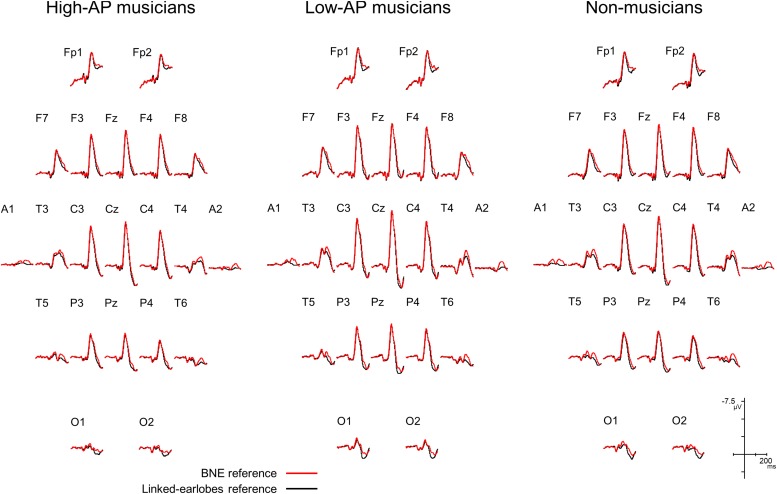
Group-averaged AEP waveforms for all EEG channels, obtained using a BNE reference (red lines) or a linked-earlobes reference (black lines). The effect of reference was evident at the temporal electrode sites where the T-complex was recorded.

We first evaluated how the use of BNE reference affected the AEP waveforms. Figure 1 compares the group-averaged AEPs for all channels, which were obtained using the BNE reference and the linked-earlobes reference. Substantial differences in the waveforms were observed at the temporal electrode sites. This was an expected result, because the radially oriented dipoles of the T-complex would produce electrical potentials at the earlobe electrodes (Wolpaw and Wood, 1982), which was also confirmed in our BNE-referenced data. When the linked-earlobes reference was used, the potentials at the earlobes were subtracted from the AEPs in the other channels to alter the wave morphologies. Specifically, the T-complex obtained with the linked-earlobes reference was artifactually reduced in amplitude when compared to that obtained with the BNE reference.

**FIGURE 2:**
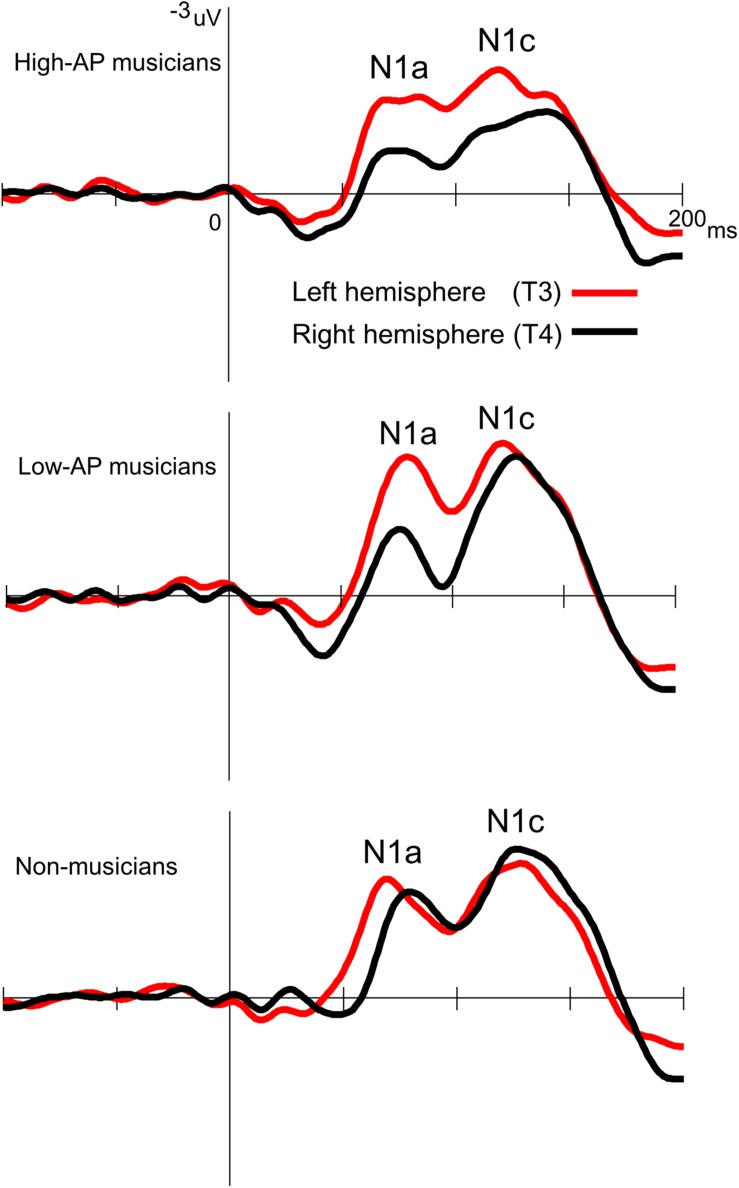
Group-averaged waveforms of the T-complex at the temporal electrodes, obtained using the BNE reference.

**FIGURE 3:**
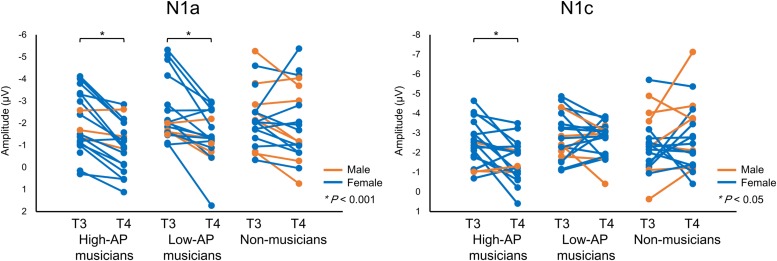
The N1a and N1c amplitudes for all individual participants. Within-subject data are connected with lines. An asterisks (^*^) denotes a statistically significant difference (*P* < 0.05).

Accordingly, we used the BNE reference to evaluate the left–right asymmetry of N1a and N1c amplitudes in high-AP musicians, low-AP musicians, and non-musicians. Figure 2 plots the T-complex waveforms at the temporal electrode sites, where they were maximal. Figure 3 shows the N1a and N1c amplitudes for all individual subjects. Four main findings were obtained.

First, only the high-AP musicians showed a left-dominant asymmetry in the N1c amplitude (Figures 2, 3). The two-way ANOVA revealed a significant group × hemisphere interaction [*F*(2,54) = 3.4, *P* = 0.042] and follow-up one-way ANOVAs indicated a significant hemispheric asymmetry in high-AP musicians, [*F*(1,54) = 6.4, *P* = 0.014] but not in low-AP musicians [*F*(1,54) = 0.3, *P* = 0.604] or in non-musicians [*F*(1,54) = 1.3, *P* = 0.264].

**FIGURE 4:**
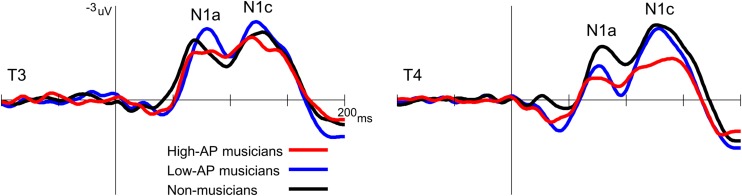
Group comparison of the T-complex amplitudes over the left (T3) and right (T4) hemispheres. At T4, the N1c was significantly smaller in the high-AP musicians than in the low-AP musicians or in the non-musicians.

Second, the left-dominant asymmetry of N1c in high-AP musicians was due to a diminution of the right N1c rather than enhancement of the left N1c (Figure 4). When the N1c amplitude was analyzed using a one-way ANOVA with the group as a factor, the main effect was significant at the right temporal electrode [T4, *F*(2,54) = 5.1, *P* = 0.009] but not at the left temporal electrode [T3, *F*(2,54) = 0.8, *P* = 0.469]. *Post hoc* analyses at T4 indicated that the right N1c amplitude was significantly smaller in the high-AP musicians than in the low-AP musicians (*P* = 0.033, Bonferroni corrected) and non-musicians (*P* = 0.017, Bonferroni corrected).

Third, both high-AP musicians and low-AP musicians showed a left-dominant asymmetry in the N1a amplitude (Figures 2, 3). The two-way ANOVA revealed a significant group × hemisphere interaction [*F*(2,54) = 3.3, *P* = 0.043], indicating that the effect of the hemisphere varied between the groups. Follow-up one-way ANOVAs indicated a significant main effect of the hemisphere in high-AP musicians [*F*(1,54) = 18.4, *P* < 0.001] and low-AP musicians [*F*(1,54) = 19.0, *P* < 0.001] but not in non-musicians [*F*(1,54) = 1.3, *P* = 0.251].

The above analyses were conducted by treating AP as a categorical variable. Considering that AP is a graded trait (Miyazaki, 1990; Bermudez and Zatorre, 2009), we additionally performed multiple regression analyses in which the N1c amplitudes were evaluated with respect to the continuous independent variables of AP test score (AP) and years in music training (Years). As a result, the model was a significant predictor of N1c at the right temporal electrode site T4 [*F*(2,35) = 7.1, *P* = 0.003]: The variable AP contributed significantly to the model (β = 0.562, *t*(35) = 3.8, *P* = 0.001), but Years did not (β = -0.123, *t*(35) = 0.8, *P* = 0.417). Regarding the left N1c at T3, the model was not a significant predictor of its amplitude (*F*(2,35) = 1.7, *P* = 0.206), with an *R*^2^ of 0.086: The contribution of AP was not significant (β = 0.308, *t*(35) = 1.8, *P* = 0.078), and the contribution of Years was also not significant (β = -0.103, *t*(35) = 0.6, *P* = 0.549). These results were consistent with the above ANOVA findings regarding the N1c.

No statistically significant effect was observed for the N1b amplitude. The one-way ANOVA with the group as a factor revealed that the main effect was not significant [Cz, *F*(2,54) = 1.7, *P* = 0.195].

One limitation of the experiment was that there were fewer male participants than female participants. Nevertheless, there were no apparent gender effects on the above findings, as could be appreciated in Figure 3.

## Discussion

The present study focused on the left–right asymmetry of the T-complex to investigate hemispheric asymmetry of the auditory cortical functions underpinning AP. Compared to musicians who had low levels of AP, musicians with high levels of AP showed a greater left-dominant asymmetry of N1c amplitude. This represented AP negativity, which has previously been described as an electrophysiological marker of AP (Itoh et al., 2005). Additionally, the use of the BNE reference in this experiment revealed that the left-dominance of N1c was caused by a reduction of N1c amplitude in the right hemisphere rather than enhancement in the left hemisphere. This AP-specific effect on N1c was distinguishable from the more general effect of music training, which manifested as a left-dominant asymmetry of N1a.

The N1c response to pure-tone stimuli is typically recorded at temporal electrode sites with no left-right asymmetry or with right-dominance in a normal population (Woods, 1995; Pang and Taylor, 2000). This well-known property of N1c was confirmed in non-musicians and musicians with low levels of AP. By contrast, only musicians with high levels of AP showed a left-dominant N1c, which closely resembled AP negativity (Itoh et al., 2005) in terms of polarity (negative), amplitude (approximately 2 μV), scalp distribution (left temporal), and latency (100–200 ms). The novel finding of this experiment was that the left-dominant asymmetry of N1c (or AP negativity) in the high-AP musician group was caused by a diminution of the right N1c, rather than enhancement of the left N1c. A distinct methodological advantage of the current experiment is that a non-cephalic reference was used. The present recording (Figure 1) showed that the earlobe electrodes were clearly activated by the T-complex as predicted, as the sources were radially oriented in the temporal lobes (Wolpaw and Wood, 1982). Therefore, it was likely that the use of the linked-earlobes reference in Itoh et al. (2005) altered the N1c amplitudes measured on the temporal scalp electrodes. We therefore recommend the use of non-cephalic references for future recordings of AP negativity.

Notably, the right N1c was diminished in musicians with high levels of AP. The sources of N1c have been estimated in the secondary or higher auditory cortex of the superior temporal gyrus (Scherg et al., 1989; Woods, 1995; Shahin et al., 2003). Thus, although scalp AEP amplitudes are affected by many factors including source location and orientation, one possible interpretation of our finding is that the pitch stimulus activated a smaller number of right auditory cortical neurons in the high-AP musician group than in the low-AP musician group. This hypothesis is in line with previous findings that the volume of the right planum temporale is smaller in AP possessors than in non-possessors (Schlaug et al., 1995; Keenan et al., 2001; Wilson et al., 2009), although Wengenroth et al. (2014) have reported an increased right auditory cortical volume in AP possessors, and Zatorre et al. (1998) have found an increased left planum polare volume in AP possessors. Considering that AP is typically acquired by early musical training, the functional and anatomical features of the right temporal lobe in AP possessors might be established in the course of brain maturation during which AP is acquired. In typical brain maturation, the N1c evoked by speech sounds gradually decreases in amplitude over the right hemisphere but not over the left hemisphere, commencing around the age of 7 years (Pang and Taylor, 2000). Moreover, the decrease in right N1c amplitude might be correlated with normal language development because the right N1c amplitude for speech sounds is apparently larger in children with language difficulties than in normal children (Groen et al., 2008). Because the core function of AP, or pitch labeling, is essentially verbal (Itoh et al., 2003), a common AEP component (i.e., right N1c) can reasonably be assumed to index both AP and language.

Two major hypotheses have been proposed regarding the neural mechanisms for AP: the early categorical perception hypothesis (Siegel, 1974) and the late labeling hypothesis (Levitin and Rogers, 2005). The early categorical perception hypothesis posits that the pitch labeling function of AP is subserved by some specialized neural circuitries in the auditory areas (Hirata et al., 1999; Itoh et al., 2005; Schulze et al., 2013), which is supported by anatomical findings that the auditory cortical structures in musicians with AP are organized differently from musicians without AP (Schlaug et al., 1995; Keenan et al., 2001; Wilson et al., 2009; Wengenroth et al., 2014; Kim and Knösche, 2016, 2017). By contrast, the late labeling hypothesis (also called the two-component model of AP) proposes that the labeling function of AP is subserved by late stages of cortical processing that occur outside the auditory areas (Zatorre et al., 1998; Levitin and Rogers, 2005). Specifically, the dorsolateral prefrontal cortex has been identified as the core brain structure that associates pitches with their names (Zatorre et al., 1998). This hypothesis is supported by studies that have found enhanced anatomical and functional connectivity between the auditory areas and the frontal lobe (Oechslin et al., 2010a; Elmer et al., 2015).

Previous AEP and event-related potential (ERP) experiments on this topic have yielded mixed results. An observation of the effect of AP on the late components of AEP/ERP (e.g., >200 ms), together with an absence of such effect on earlier components (e.g., <200 ms), is sometimes taken as evidence for the late labeling hypothesis (Tervaniemi et al., 1993; Rogenmoser et al., 2015). However, several AEP/ERP experiments have demonstrated AP-related differences in the early stages of auditory cortical processing (Hirata et al., 1999; Itoh et al., 2005; Wu et al., 2008; Coll et al., 2019), and our present results corroborate these findings.

In addition to the main findings regarding the neural correlates of AP, a left-dominant asymmetry of N1a was fortuitously identified as a novel electrophysiological marker of music training. Music training is known to enhance the tangentially oriented component of N1 (or its magnetic counterpart N1m) elicited by tone stimuli, which typically has a left-dominant distribution at a peak latency of approximately 100 ms (Pantev et al., 1998; Kuriki et al., 2006; Baumann et al., 2008; Itoh et al., 2012). Our result adds to these findings by revealing that the well-known enhancement of N1 (N1m) in musicians is preceded by left-dominant neural activities at an earlier stage of auditory cortical processing at approximately 80 ms. This is in line with the findings that plastic changes due to music training occur throughout the auditory pathway, including the subcortical (Musacchia et al., 2007) and cochlear (Bidelman et al., 2016) levels of processing.

## Conclusion

In conclusion, two main findings were obtained: (1) a left-dominant N1c (at approximately 130 ms) indexes AP, and (2) a left-dominant N1a (at approximately 80 ms) indexes music training. We conclude that the faculties for music and AP are both accompanied by a left-dominant hemispheric specialization of auditory cortical functions, but that they affect distinct stages of pitch processing in the human auditory cortex.

## Data Availability

The raw data supporting the conclusions of this manuscript will be made available by the authors, without undue reservation, to any qualified researcher.

## Ethics Statement

This human study was carried out in accordance with the recommendations of the Ethical Guidelines for Medical and Health Research Involving Human Subjects (Ministry of Education, Culture, Sports, Science, and Technology; Ministry of Health, Labor, and Welfare) with written informed consent from all subjects. All subjects gave written informed consent in accordance with the Declaration of Helsinki. The protocol was approved by the Internal Review Board of the University of Niigata.

## Author Contributions

MM and KI conceived and designed the study. MM and KI conducted the experiments. MM analyzed the data. All authors wrote the manuscript.

## Conflict of Interest Statement

The authors declare that the research was conducted in the absence of any commercial or financial relationships that could be construed as a potential conflict of interest.
